# Understanding the Impact of Sustainable Pharmaceutical Packaging on the Chemical Stability of Silodosin

**DOI:** 10.3390/pharmaceutics17121548

**Published:** 2025-11-30

**Authors:** Celia Visa, Roi Rodriguez, Ángela Rincón, Soledad Peña, Dolores Remedios Serrano, Juan José Torrado

**Affiliations:** 1 Cloverty, Alicante 8-10, 28500 Arganda del Rey, Madrid, Spain; celia.visa@cloverty.com (C.V.); roi.rodriguezf@gmail.com (R.R.); angela.rincon@cloverty.com (Á.R.); soledad.pena@cloverty.com (S.P.); 2 Pharmaceutics, School of Pharmacy, Complutense University of Madrid, Ramon y Cajal, 28040 Madrid, Spain; 3 Instituto Universitario de Farmacia Industrial, Faculty of Pharmacy, Universidad Complutense de Madrid, 28040 Madrid, Spain

**Keywords:** silodosin, sustainable packaging materials, degradation kinetics, WVTR, soft capsules, impurities

## Abstract

**Background/Objectives**: Silodosin (SLD) is a selective α1A-adrenoceptor antagonist used in the treatment of benign prostatic hyperplasia. Bioequivalence failures have been reported for hard capsule formulations, largely due to the effect of disintegrant excipients, making soft capsules a promising alternative dosage form. This study investigated the stability of SLD soft capsules stored in two different packaging materials, PVC/PVDC and AquaBa^®^. **Methods**: Storage temperatures at 25 °C/60%, 30 °C/65% RH, 30 °C/75% RH, and 40 °C/75% RH, and sampling were performed according to the International Council for Harmonisation (ICH) stability conditions. Assays were performed by HPLC and UV, and mass detection. **Results**: Degradation analysis revealed that temperature played a critical role in SLD degradation and the formation of its primary degradation products, dehydrosilodosin and impurity 1. **Conclusions**: AquaBa^®^ demonstrated superior protective properties compared to PVC/PVDC, preserving SLD content above 95% for over 12 months under 25 °C and 30 °C conditions while limiting the formation of degradation products. Nevertheless, impurity 1 exceeded its ICH Q3B (R2) specification limit (0.3%) after six months under all conditions tested, suggesting a critical interaction between SLD and excipients such as Capryol^®^ 90. Kinetic modeling confirmed first-order degradation kinetics for both dehydrosilodosin and impurity 1, with a faster degradation rate observed in PVC/PVDC blisters. These findings highlight the critical role of packaging in pharmaceutical stability. While AquaBa^®^ emerges as the preferred option for SLD soft capsules, formulation optimization remains necessary to limit impurity formation, extend shelf life, and ensure regulatory compliance.

## 1. Introduction

Silodosin (SLD) is a selective α1-adrenergic receptor antagonist widely prescribed for the treatment of benign prostatic hyperplasia [1]. According to the Biopharmaceutics Classification System (BCS), SLD is a class III compound with high solubility and low permeability [2]. Recent studies have reported bioequivalence failures in hard capsule formulations due to the influence of disintegrant excipients [2]. Soft capsules, which generally provide faster release and absorption, therefore represent a promising dosage form for SLD administration [3].

However, the stability of SLD in soft gelatine capsules can be challenging and requires a detailed drug: excipient compatibility study [4]. In our study, binary combinations of SLD with several excipients were formulated. Subsequently, these formulations were subjected to thermal degradation conditions for different periods. The formulation containing a binary combination of SLD and propylene glycol monocaprylate type II (Capryol^®^ 90) led to the formation of an unknown impurity. During conventional stability studies [5], this unknown impurity, previously detected in the compatibility studies, grew above the specification limits set by the ICH Q3B (R2) [6]. To enhance the pharmaceutical stability of SLD in soft capsules, the impact of different pharmaceutical packings was investigated.

Pharmaceutical packaging plays a crucial role in drug stability by protecting medications from moisture, light, and other environmental factors [7]. AquaBa^®^ and PVC (Polyvinyl Chloride) are two materials used for pharmaceutical packaging, but they have significant differences in composition, barrier properties, and sustainability. Aquaba^®^ is a biodegradable and PVC-free packaging material developed as a sustainable alternative to traditional plastics in the pharmaceutical industry [8]. In contrast, PVC (Polyvinyl Chloride) is a synthetic polymer derived from vinyl chloride. PVC is widely used in pharmaceutical packaging due to its affordability and versatility, but its environmental impact has led to increased regulatory scrutiny [9,10].

One of the key factors in choosing a packaging material is its barrier properties, particularly against moisture and oxygen, which can affect drug stability [11]. Pure PVC has poor moisture barrier properties, which is why it is often combined with PVDC (Polyvinylidene Chloride) in pharmaceutical applications to enhance protection. AquaBa^®^, however, offers an improved barrier against moisture and oxygen, similar to PVC/PVDC multilayer materials, making it a viable alternative without the need for vinyl chloride.

Sustainability is a growing concern in pharmaceutical packaging, and materials like AquaBa^®^ are gaining attention due to their lower environmental impact. AquaBa^®^ is a sustainable alternative that eliminates the environmental issues associated with PVC, particularly the release of chlorine and dioxins during production and disposal. Meanwhile, PVC is facing increasing restrictions in the industry due to its environmental impact, leading many pharmaceutical companies to seek more sustainable alternatives.

PVC remains a widely used material in blister packs, especially in combination with PVDC to improve moisture barrier properties. However, AquaBa^®^ is emerging as a more sustainable alternative, offering similar performance to PVC/PVDC blister packs with significantly lower environmental impact. However, more studies are required to investigate its impact on active pharmaceutical ingredients’ stability.

The water vapour transfer rate (WVTR) measures the amount of water vapor that permeates through a material over a specific period, typically expressed in grams per square meter per day (g/m^2^·day) [12]. Lower MVTR values indicate better moisture barrier properties. For uncoated PVC (250 µm thickness), the WVTR is approximately 3.1 g/m^2^·day at 38 °C and 90% relative humidity, while for PVC with 40 g/m^2^ with a PVDC Coating, the WVTR is 0.65 g/m^2^·day [13]. In contrast, AquaBa^®^ combines proprietary calendered PVC film with a SuperB coating, resulting in an ultra-high water vapor and oxygen barrier with WVTR ranging from 0.06 to 0.11 g/m^2^·day (for a 250 µm film) and an oxygen transmission rate ranging from 0.07 to 0.13 cm^3^/m^2^/day/bar [14].

If sustainability and high-barrier properties without PVC are priorities, AquaBa^®^ could be an excellent choice for pharmaceutical packaging, overcoming stability barriers for certain products such as soft capsules. This work, therefore, aims to compare the chemical stability of SLD soft capsules packaged in AquaBa^®^ and in conventional PVC/PVDC blisters under ICH storage conditions, with a particular focus on degradation kinetics and impurity formation. By integrating excipient compatibility studies, stability testing, and kinetic modeling, this study provides new insights into the stability challenges of SLD formulations.

## 2. Materials and Methods

### 2.1. Materials

SLD was received from Tyche Industries Limited (Hyderabad, India), while propylene glycol mono-caprylate type II (Capryol^®^ 90) was obtained from Gattefossé (Saint-Priest, France). HPLC-grade methanol and acetonitrile were obtained from Scharlab (Barcelona, Spain) and PanReac (Castellar del Vallés, Spain), respectively. Ortho Phosphoric acid (88% purity) was purchased from PanReac (Castellar del Vallés, Spain), and formic acid was obtained from Honeywell (Charlotte, NC, USA). Purified demineralized water, purification system Elga Purelab Model PC110COBM1 (High Wycombe, UK), was used. Ester and amide forms of SLD were synthesized and supplied by Galchimia company (Fonte Díaz, Spain). Aconditioning material, PVC/PVDC (PVC 250 µm + PVDC 120 g/m^2^, white opaque) from Enteco Pharma, S.L. (Móstoles, Spain), and a multilayer PVC-PVDC film (AquaBa^®^ DX 200, PVC 250 µm + PVDC 200 g/m^2^) supplied by Liveo Research GmbH (Basel, Switzerland) were used as primary packaging material.

### 2.2. Excipient Compatibility Studies

The sample preparation consisted of single ingredients and binary and ternary combinations of SLD with the different excipients of the formulation, including Capryol^®^ 90 as well as Lauroyl macrogol-32 glycerides and Butylhydroxytoluene (BHT), as shown in Table 1. The aqueous solubility of SLD in aqueous pH media is lower than 0.3 mg/mL [2]. Therefore, to achieve a correct dosing in soft gelatin capsules, a mixture of cosolvents and surfactant excipients is required. Lauroyl macrogol-32 glycerides is a nonionic, water-dispersible surfactant, which was combined with Capryol^®^ 90, which is a nonionic, water-insoluble surfactant. Moreover, antioxidant BHT was also included as a potential excipient in the formulation. Excipients/SLD ratio was selected while maintaining the ratio of the final formulation. Samples were exposed to thermal degradation in a heater set at 70 °C, and SLD-related substances were evaluated by HPLC at 0, 7, and 30 days.

### 2.3. Long-Term ICH Stability Study

The most compatible excipient combination was identified based on dissolution of SLD and chemical stability under thermal stress, considering the percentage of SLD remaining and the levels of related impurities formed at 0, 7, and 30 days. The combination of Capryol^®^ 90 and Lauroyl macrogol-32 glycerides supplemented with BHT seems related to the best dissolution and lowest SLD degradation, and it was therefore selected for further formulation development. This combination was used to obtain SLD soft capsules, which were packed in PVC/PVDC or AquaBa^®^ materials and then subjected to conventional stability tests, following the ICH Q1A (R2) indications [5]. Samples were stored at different conditions: 25 °C/60% RH, 30 °C/65% RH, and 40 °C/75% RH. Samples were collected at different time points (1, 3, and 6 months), and both SLD and impurities were quantified using a validated indicating stability HPLC method.

### 2.4. HPLC Quantification of SLD and Related Substances

The impurity detection and quantification analysis were carried out using an HPLC Shimadzu Nexera^®^ Series LC-40 (Shimadzu, Kyoto, Japan), consisting of an SPD-M40 photodiode array detector. Separation was achieved on a Nouryon Kromasil^®^ C8 column (4.6 mm × 250 mm, 5 µm). The mobile phase flow rate was fixed at 1.0 mL/min. The mobile phase was a mixture of mobile phases A and B in gradient mode. Phase A comprised 0.01% orthophosphoric acid (88% purity) with a pH of 2.85. Acetonitrile was used as mobile phase B. The initial conditions of the gradient program were 78% of B, reaching 15% of B, and finally returning to the initial conditions in a gradient flow condition described in Table 2.

A constant temperature of 25 °C was maintained inside the column. The detection wavelength was 225 nm, and the injection volume was 20 µL. The diluent used for the sample preparation was HPLC-grade methanol.

### 2.5. Stability Modeling

The degradation rate of SLD was calculated by fitting the percentage of degradants (dehydrosilodosin and impurity 1) at different time points to several degradation kinetic equations (zero order, first order, second order, Avrami, and diffusion), and the best-fitted degradation kinetic model was selected [15]. The model with the highest R^2^ was selected, and the slope, which is equivalent to the degradation constant at this condition, was determined [16,17]. In zero-order kinetics, the degradation rate is unchanged as the amount of drug substance decreases. However, it is relatively rare to find this type of reaction. Apparent zero-order kinetics typically reflect underlying first- or second-order reactions that appear linear when observed over a limited time window, before the degradation rate slows as the drug is consumed [18].

Mathematical modelling was based on the modified Arrhenius equation (Equation (1)) [16,19]. The classical Arrhenius equation is good for predicting API stability from liquid dosage forms such as solutions:

(1) $$LnK=LnA-\;\frac{Ea}{RT}$$

where *K* is the chemical reaction rate; *A* is a constant referred to as the “preexponential factor”; *Ea* is the activation energy of the reaction, typically measured in Kcal/mol, that describes the “temperature sensitivity” of the drug; *R* is the universal gas constant, whose value is 1.987 cal/K·mol; and *T* is the absolute temperature expressed in Kelvin degrees [17]. In these studies, the specification limit for dehydrosilodisin was 0.6% and for impurity 1 was 0.3% according to the European Pharmacopeia [20]; hence, high percentages of degradation were not relevant, and prolonged time points were not taken into consideration.

### 2.6. Permeability of Packing Materials

The permeability of the PVC/PVDC and AquaBa^®^ blisters was indirectly evaluated by monitoring the weight variation in SLD soft capsules during storage at the conditions described in Section 3.3 (40 °C/75% RH, 30 °C/65% RH, and 25 °C/60% RH). Capsules were weighed before packaging and after 1, 3, and 6 months using a precision analytical balance (Ohaus Pioneer, Parsippany, NJ, USA). The increase in capsule weight over time was interpreted as an indicator of water vapor ingress through the blister film, in line with previously reported gravimetric approaches for assessing packaging barrier properties [17,18]. This indirect evaluation correlates with the moisture uptake of the dosage form and therefore reflects the relative permeability of the packaging systems. Moisture content analyses of selected samples confirmed the same trend, validating the gravimetric results.

### 2.7. Statistics

At least three samples were assayed for each experimental condition. Data treatments, ANOVA, and Student’s two-tailed paired t-test were performed with Excel (Office 365, Microsoft).

## 3. Results and Discussion

### 3.1. HPLC Validation

**Figure 1:**
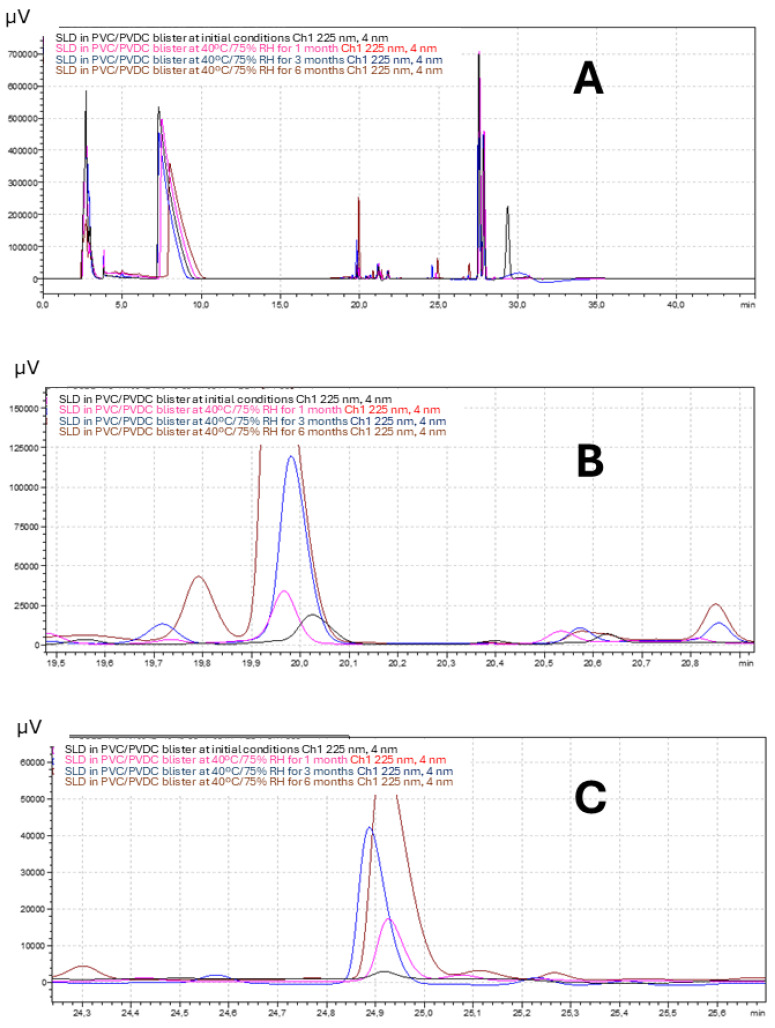
(**A**) Superposition of chromatograms corresponding to the SLD formulation packed in PVC/PVDC blister at initial conditions (black) and after storage at 40 °C/75% RH for 1 (pink), 3 (blue), and 6 (brown) months. Retention times (RT) for SLD, dehydrosilodosin, and impurity 1 were approximately 8, 20, and 25 min. (**B**,**C**) show a magnification of the peaks corresponding to dehydrosilodosin, and impurity 1, respectively.

Figure 1A shows a representative superposition of chromatograms corresponding to the SLD formulation packed in PVC/PVDC blister at initial conditions (black) and after storage at 40 °C/75% RH for 1 (pink), 3 (blue), and 6 (brown) months. Retention times for SLD, dehydrosilodosin, and impurity 1 were approximately 8, 20, and 25 min. Figure 1B,C show a magnification of the peaks corresponding to dehydrosilodosin, and impurity 1, respectively.

The method was validated. Linearity was studied between 50% and 150% of the specification limit (0.13% for unknown impurities and 0.31% for known impurities of SLD’s nominal concentration, corresponding with 1.2 and 3.7 µg/mL). The limit of detection and quantification were 0.10 and 0.30 µg/mL, respectively. The correlation coefficient was 1.0, and repeatability values (n = 6) were 95.2% with an RSD of 5.3%. The accuracy at 1.2 and 3.7 µg/mL was 92.5% and 96.8%, respectively. The intermediate precision (RSD) for a 2.5 µg/mL concentration estimated on two different days by two analysts was 5.5%.

### 3.2. Excipient Compatibility Studies

The results of the excipient compatibility study are reported in Table S1 in the supplementary material section. The most relevant is that impurity 1 was found in the presence of SLD and Capryol^®^ 90 combinations, which can be attributed to an interaction between SLD and this excipient. Furthermore, the levels of impurity 1 detected after 0, 7, and 30 days of degradation at 70 °C are similar in all the samples containing Capryol^®^ 90, so it was assumed that the formation of this impurity is not increased by the presence of the rest of the excipients of the formulation.

**Figure 2:**
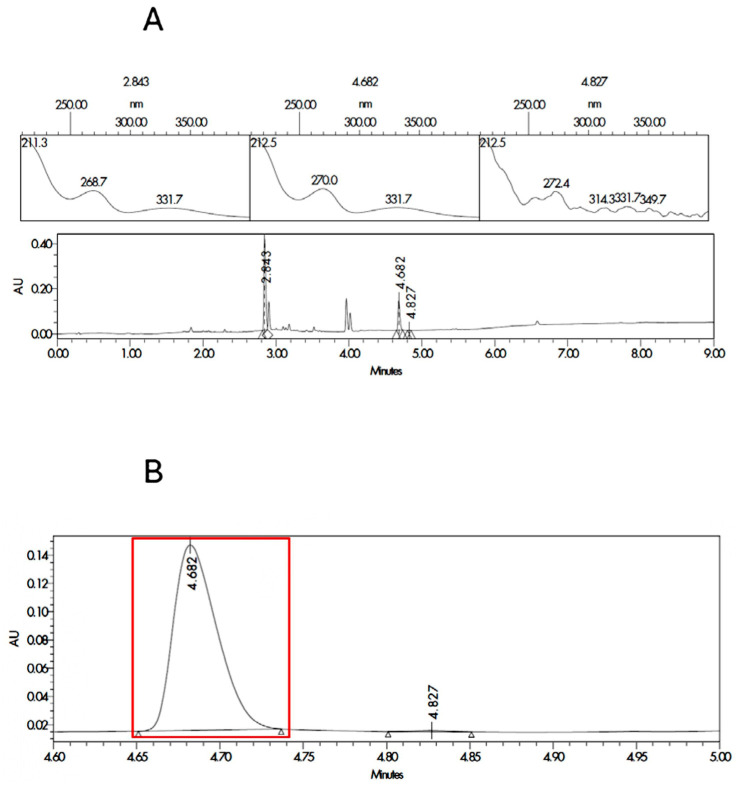
Spectrum index plots and PDA spectrum (**A**) for the peaks corresponding to SLD (RT 2.8 min and area 695.1 µV·s) and impurity 1 enriched with the ester synthesized form (RT 4.7 and area 237.2 µV·s). Magnification of the chromatogram at the times related to the impurity 1 (**B**) for the sample corresponding to degraded SLD contaminated with the ester form is marked in red.

**Figure 3:**
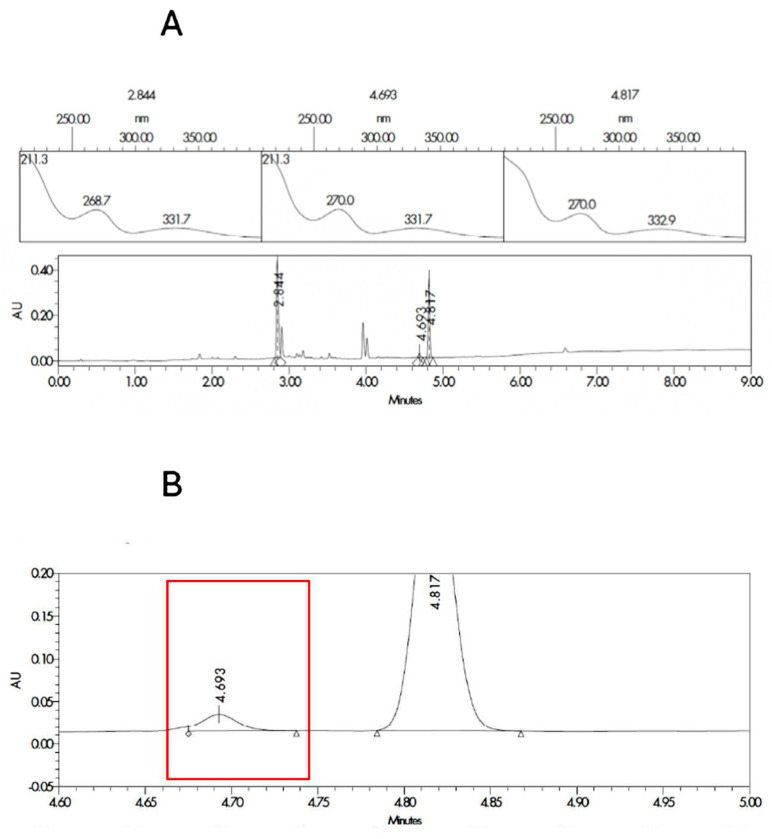
Spectrum index plots and PDA spectrum (**A**) for the peaks corresponding to SLD (RT 2.8 min and area 748.5 µV·s), impurity 1 (RT 4.7 min and area 29.3 µV·s), and amide synthesized form (RT 4.8 and area 512.2 µV·s). Magnification of the chromatogram at the times related to the impurity 1 (**B**) marked in red for the sample corresponding to degraded SLD contaminated with the amida form.

The two most probable structures for impurity 1 are the ester or the amide, and they are described in Figure S1 in the supplementary material. In order to ensure the identification of impurity 1, degraded samples of SLD containing propylene glycol and Capryol^®^ 90 were contaminated with the synthesized impurities. Samples were analyzed, and the results for the spectrum index plots and PDA spectrum for the ester and Amida forms are shown in Figure 2 and Figure 3, respectively. The coinjection of the SLD degraded sample with the Ester form synthesized matched the retention time of impurity 1 detected in the excipient compatibility study.

### 3.3. Long-Term ICH Stability Study

**Figure 4:**
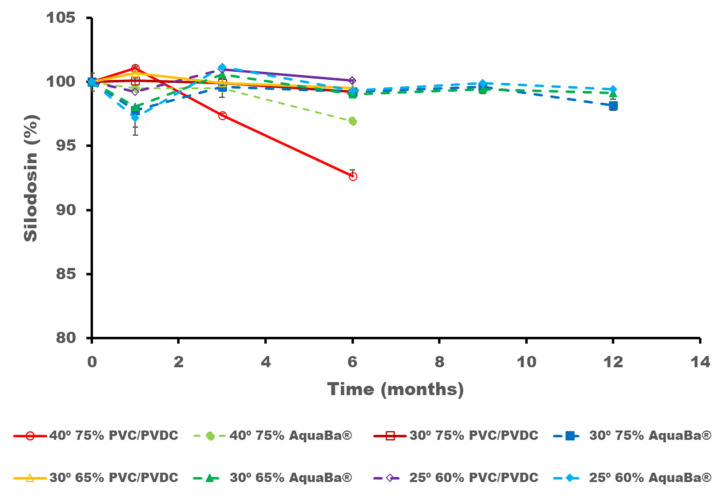
SLD content expressed in initial percentage remaining (%) after storage at different conditions and times.

During the conventional ICH stability studies, the degradation of SLD as well as the formation of dehydrosilodosin and impurity 1 were detected and quantified. A significant degradation for SLD occurred at 40 °C/75% RH when stored in PVC/PVDC blisters compared to AquaBa^®^ after three months of storage (*p* < 0.05) (Figure 4). However, SLD remained above 95% when soft capsules were packaged in AquaBa^®^ blisters for over 12 months at 30 °C/65% RH and 25 °C/60% RH conditions. After 6 months of storage, it was clear that AquaBa^®^ provides better protection for SLD than the conventional PVC/PVDC packing, so no further assays were performed with the PVC/PVDC samples. Data from Figure 4 is also reported in Table S2 of the supplementary section.

**Figure 5:**
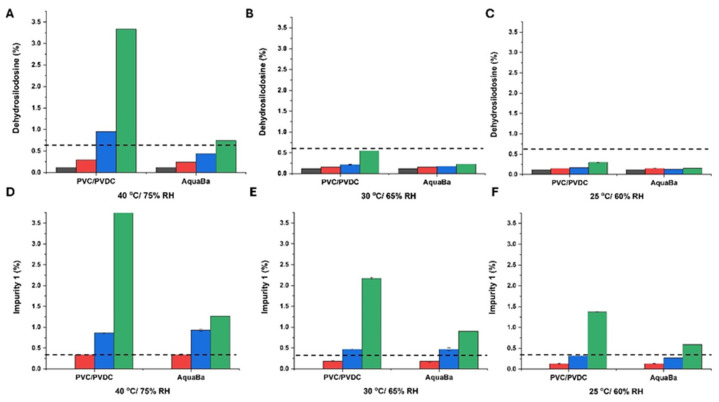
Formation of dehydrosilodosin (**A**–**C**) and impurity 1 (**D**–**F**) at different storage conditions and times expressed as percentage ± SD. The dotted line shows the specification limit for dehydrosilodosin (<0.6%) and impurity one (<0.3%), respectively. Black colour refers to non-degraded samples (0 months), red colour refers to samples after 1 month storage, blue colour refers to samples after 3-month storage, and green colour refers to samples after 6-month storage.

Regarding the formation of degradation products, dehydrosilodosin and impurity 1, temperature and packing material played a critical role (Figure 5). The higher the temperature, the higher the percentage of degradation products. Also, AquaBa^®^ demonstrated a superior capacity to PVC/PVDC to preserve the integrity of SLD, minimizing the degradant formation.

However, none of the soft capsule formulations exhibited an optimal stability profile. Even though the levels of SLD remained above the specification limit (95%) over 12 months at 25 °C/60% RH and the dehydrosilodosin levels were kept below 0.6%, the percentage of impurity 1 was well above the specification limit (0.3%) at 6 months at all the ICH conditions tested. This highlights the importance of compatibility studies to predict potential interactions amongst API and excipients.

**Figure 6:**
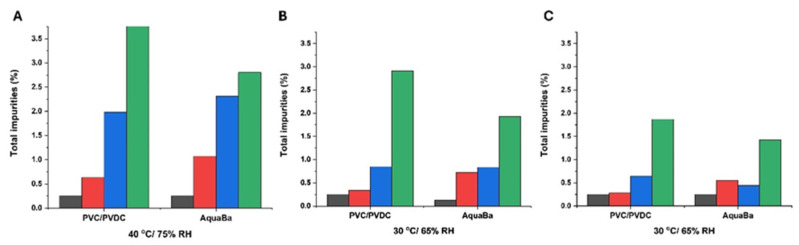
Total impurities formed using AquaBa^®^ and PVC/PVDC packing materials at different storage conditions and times (**A**–**C**). Black colour refers to non-degraded samples (0 months), red colour refers to samples after 1 month storage, blue colour refers to samples after 3-month storage, and green colour refers to samples after 6-month storage.

**Figure 7:**
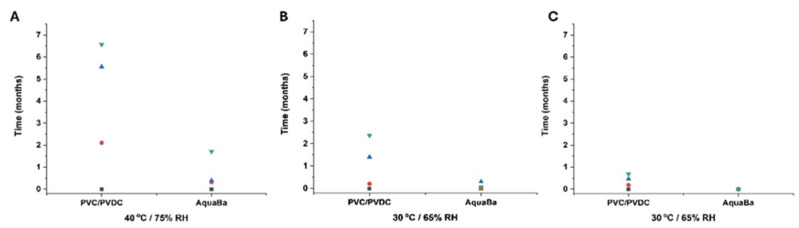
Relative total weight increase in capsules using AquaBa^®^ and PVC/PVDC packing materials at different storage conditions and times (**A**–**C**). Black colour refers to non-degraded samples (0 months), red colour refers to samples after 1 month storage, blue colour refers to samples after 3-month storage, and green colour refers to samples after 6-month storage.

A total of five impurities were found in agreement with results previously reported by other authors [21]. The total impurity content was also determined and exhibited a similar profile to impurity 1. AquaBa^®^ was more effective than PVC/PVDC in preserving the integrity of SLD in soft capsules at 6 months (Figure 6). This superior effectiveness of AquaBa^®^ over PVC/PVDC was attributed to its lower MVTR. As observed in Figure 7, the weight increase in soft capsules stored in AquaBa^®^ blisters was significantly lower (*p* < 0.05) at 40 °C/75% RH and 30 °C/65% RH, while no significant differences were observed at 25 °C/60% RH. The lower weight gain indicates reduced moisture uptake by the capsules, consistent with a lower effective WVTR of the AquaBa^®^ packaging. By limiting water ingress into the pack, AquaBa^®^ reduces the amount of free water available for hydrolytic processes, thereby mitigating hydrolytic degradation and contributing to improved formulation stability under elevated temperature/humidity conditions.

When modeling the degradation kinetics of dehydrosilodosin and impurity 1, several kinetic models (zero-order, first-order, second-order, Avrami, and diffusion-controlled) were evaluated. The selection of the first-order kinetic model was based on statistical goodness-of-fit criteria, including the highest coefficient of determination (R^2^ > 0.95 for most conditions), and the visual agreement between experimental and predicted profiles (Table 3 and Table 4). These criteria indicate that in our experimental conditions the first-order model provides the most accurate representation of the degradation behavior of both compounds across all tested conditions. However, it should be noted that a more prolonged stability study to observe a higher degradation of the products should provide a more accurate adjustment to kinetic models.

**Figure 8:**
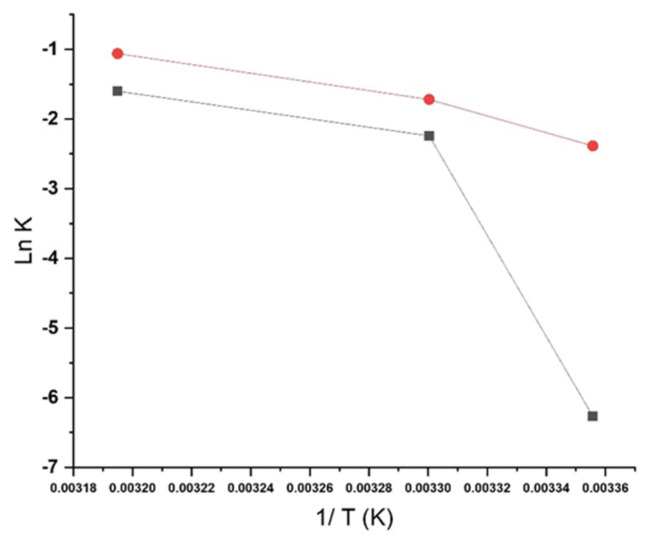
Degradation constants (K) from first-order kinetics are represented at different temperatures for dehydrosilodosin Key: -■- AquaBa^®^, -●- PVC/PVDC.

No statistically significant differences were found in the overall degradation kinetics between soft capsules stored in PVC/PVDC and AquaBa^®^ blisters. However, compared to AquaBa^®^ (with a Ea = 51.5 Kcal/mol), a lower activation energy (Ea) was observed for dehydrosilodosin in PVC/PVDC blisters (Ea = 15.8 Kcal/mol) (Figure 8), suggesting a higher susceptibility to hydrolytic degradation under humid conditions. This observation is consistent with the lower moisture vapor transmission rate (MVTR) of AquaBa^®^, indicating that reduced moisture ingress mitigates hydrolysis and contributes to greater formulation stability. In contrast, impurity 1 exhibited comparable kinetic parameters in both packaging materials, implying that its formation may be influenced not only by humidity but also by temperature and excipient interactions promoting ester formation within the soft capsule matrix.

SLD was found to be labile under hydrolytic and oxidative conditions [22]. The present study evaluated the stability of SLD in soft capsules when stored in two different packaging materials: PVC/PVDC and AquaBa^®^. The results indicate that the choice of packaging material has a significant impact on the stability of SLD over time, as assessed through key parameters such as degradation profile and moisture content.

The data suggests that AquaBa^®^ provides superior protection against environmental factors that contribute to SLD degradation, particularly moisture ingress. The capsules stored in AquaBa^®^ exhibited lower levels of degradation products and maintained a higher percentage of the API content over the study period. This improved stability can be attributed to the enhanced barrier properties of AquaBa^®^, which effectively restricts moisture penetration and oxygen exposure, two key factors known to accelerate the degradation of SLD.

In contrast, the PVC/PVDC packaging demonstrated comparatively lower stability performance. The capsules stored in this material showed a greater increase in degradation products, indicating that the protective barrier offered by PVC/PVDC is less effective than AquaBa^®^. One possible explanation for this observation is the inherent permeability of PVC/PVDC to moisture and gases, which might allow for gradual environmental influence on formulation stability. Previous studies have also reported that PVDC coatings, while providing an improved barrier compared to conventional PVC, still permit some degree of moisture ingress, which could explain the observed trends [13].

Additionally, the effect of storage conditions on SLD stability was evident in both packaging types. Higher humidity and temperature conditions accelerated the degradation process, with more pronounced effects observed in the PVC/PVDC packaging. This suggests that for long-term storage, particularly in regions with high humidity, AquaBa^®^ is the best choice for maintaining the integrity of SLD soft capsules.

The formation of the ester degradation product of SLD suggests that hydrolysis may not be the only pathway for degradation, which explains why temperature also plays a significant role. Unlike hydrolysis-driven degradation pathways, esterification generally does not require significant water availability. AquaBa^®^ packing material prevents moisture, and hence, the absence of moisture’s impact suggests that the esterification occurs through a different mechanism, possibly involving interactions between SLD and excipients or degradation intermediates at elevated temperatures. Some excipients in the soft capsule formulation might undergo thermal degradation, releasing reactive species that catalyze ester formation [23].

The formation of impurity 1, related to the composition of the formulation, was observed at the beginning of the experimental work after compatibility studies at 70 °C. At that time, the long-term stability study had already started. It was thought that the study could be continued and test the effect of packing protection using impurity 1 as a reference for potential protection against instability. The results of the long-term stability study proved that AquaBa^®^ packing material was superior to PVC/PVDC not only for SLD but also for the impurities studied in the assay.

The formation of Impurity 1 appears to be associated not only with hydrolytic degradation but also with potential drug–excipient interactions within the soft capsule formulation. SLD, being susceptible to ester formation under certain conditions, may react with excipients containing hydroxyl or ester functional groups, especially in the presence of residual moisture. This suggests a possible inherent incompatibility between SLD and some commonly used solubilizers or plasticizers in soft capsule formulations.

To minimize this risk, formulation strategies such as the use of alternative solubilizers with lower reactivity (e.g., polyethylene glycol derivatives of higher molecular weight or non-ester-based surfactants), optimization of the capsule fill pH, and strict control of residual water content could be considered. These approaches would help mitigate esterification reactions leading to Impurity 1 and enhance overall formulation stability. Future work will focus on evaluating excipient compatibility through stress testing and identifying excipients that minimize degradation under accelerated conditions.

From a pharmaceutical perspective, the selection of suitable excipients and packaging material is a critical factor in drug stability and shelf-life [7]. The results of this study underscore the importance of excipient compatibility studies and adopting packaging solutions with superior barrier properties to ensure product efficacy and safety. Given the demonstrated benefits of AquaBa^®^ in preserving SLD stability, its use should be strongly considered for commercial packaging applications, particularly for formulations sensitive to moisture and oxidative degradation.

Further studies may be warranted to explore the mechanistic details of SLD degradation under varying environmental stressors and to assess the economic feasibility of adopting AquaBa^®^ packaging on a larger scale.

## 4. Conclusions

The stability study of SLD in soft capsules stored in PVC/PVDC and AquaBa^®^ blisters under ICH conditions revealed significant differences in degradation behavior and impurity formation. A key finding was the superior protective capacity of AquaBa^®^ compared to PVC/PVDC, particularly in minimizing degradation at elevated temperatures. AquaBa^®^ is a biodegradable, recyclable, and high-barrier packaging material that serves as an eco-friendly alternative to PVC/PVDC in pharmaceutical applications. While PVC remains a cost-effective and widely used option, it has limitations in permeability and environmental impact, requiring additional coating (such as PVDC or aluminum) for enhanced protection. Despite AquaBa’s^®^ superior performance in limiting degradation, neither packaging material fully prevented impurity formation. While SLD remained above 95% at 25 °C/60% RH for 12 months, impurity 1 exceeded the specification limit (0.3%) at six months across all tested conditions. This highlights the need for further formulation optimization to mitigate impurity formation and improve the overall stability profile of SLD soft capsules.

## Figures and Tables

**Table 1:** Samples were exposed to thermal degradation (70 °C) during excipient compatibility studies.

Sample Code	Component
1	SLD
2	Capryol^®^ 90
3	Lauroyl macrogol-32 glycerides
4	BHT
5	SLD + Capryol^®^ 90
6	SLD + Lauroyl macrogol-32 glycerides
7	SLD + BHT
8	SLD + Capryol^®^ 90 + Lauroyl macrogol-32 glycerides
9	SLD + Capryol^®^ 90 + Lauroyl macrogol-32 glycerides + BHT
10	Capryol^®^ 90 + Lauroyl macrogol-32 glycerides
11	Capryol^®^ 90 + Lauroyl macrogol-32 glycerides + BHT

**Table 2:** Gradient flow conditions.

Time (min)	% Mobile Phase A	% Mobile Phase B
0.01	78	22
13.00	78	22
28.00	15	85
37.00	78	22
45.00	78	22

**Table 3:** Fitting different degradation Kinetics for dehydrosilodosin and impurity 1 in PVC/PVDC expressed as R^2^ value after storage at different conditions.

Kinetic Profile	40 °C/75% RH	30 °C/65% RH	25 °C/60% RH	Average
Dehydrosilodosin				
Order 0	0.85	0.87	0.87	0.86
Order 1	1.00	0.88	0.88	0.92
order 2	0.99	0.87	0.87	0.91
Avrami	0.96	0.75	0.75	0.82
Difussion	0.90	0.85	0.80	0.85
Impurity 1				
Order 0	0.85	0.87	0.87	0.86
Order 1	0.99	0.99	0.99	0.99
order 2	0.92	0.92	0.93	0.92
Avrami	0.91	0.92	0.92	0.92
Difussion	0.85	0.85	0.85	0.85

**Table 4:** Fitting different degradation Kinetics for dehydrosilodosin and impurity 1 in AquaBa^®^ expressed as R^2^ value after storage at different conditions. * Indicates that fitting to any degradation profile was not feasible due to limited degradation.

Kinetic Profile	40 °C/75% RH	30 °C/65% RH	25 °C/60% RH	Average
Dehydrosilodosin				
Order 0	0.98	0.84	*	0.91
Order 1	0.98	0.96	*	0.97
order 2	0.99	0.92	*	0.96
Avrami	0.99	0.86	*	0.93
Difussion	0.64	0.72	*	0.77
Impurity 1				
Order 0	0.91	0.9	0.91	0.91
Order 1	0.99	0.99	0.99	0.99
order 2	0.92	0.93	0.93	0.93
Avrami	0.91	0.92	0.92	0.92
Difussion	0.85	0.85	0.85	0.85

## Data Availability

The original contributions presented in this study are included in the article. Further inquiries can be directed to the corresponding authors.

## Supplementary Materials

The following supporting information can be downloaded at: https://www.mdpi.com/article/10.3390/pharmaceutics17121548/s1, Figure S1: Chemical structures and molecular weights of SLD (A), propylene glycol monocaprylate (B), ester between SLD and octanoic acid (C), and the amide between SLD and the octanoic acid (D); Table S1: Mean results and standard deviation in parenthesis of SLD and impurity 1 for the samples during the compatibility study at 70 °C at 0, 7, and 30 days. Key: Identification of the composition of the coded samples are described in Table 1, Silodosin (SLD), Dehydrosilodosin (DSLD), Impurity 1 (Imp. 1), Detection Limit (DL), Not apply (NA); Table S2: Effect of packing material on stability of SLD (SD in parenthesis) depending on time exposition in months at the different storage conditions.
