# Supplementary material for: Understanding the Impact of Sustainable Pharmaceutical Packaging on the Chemical Stability of Silodosin
Source: Pharmaceutics. 2025 Nov 30;17(12):1548. doi: 10.3390/pharmaceutics17121548 (PMC12736067; doi:10.3390/pharmaceutics17121548)
Supplement: Supplementary file 1 [file pharmaceutics-17-01548-s001.zip › pharmaceutics-3972547-supplementary.pdf]

Excipient compatibility study

Table S1 shows the results of the compatibility study.

Table S1. Mean results and standard deviation in parenthesis of SLD and impurity 1 for the samples during the compatibility study at 70 ºC at 0, 7, and 30 days. Key: Identification of the composition of the coded samples are described in Table 1, Silodosin (SLD), Dehydrosilodosin (DSLD), Impurity 1 (Imp. 1), Detection Limit (DL), Not apply (NA).

Sample code	0			7			30		
	SLD (%)	DSLD (%)	Imp. 1 (%)	SLD (%)	DSLD (%)	Imp. 1 (%)	SLD (%)	DSLD (%)	Imp. 1 (%)
1	100.0	0.06	< DL	100.0 (0.3)	0.07	< DL	99.7 (8.9)	0.12	< DL
2	NA	< DL	< DL	NA	< DL	< DL	NA	< DL	< DL
3	NA	< DL	< DL	NA	< DL	< DL	NA	< DL	< DL
4	NA	< DL	< DL	NA	< DL	< DL	NA	< DL	< DL
5	100.0	0.06	< DL	95.5 (1.7)	1.85	0.49	89.7 (4.6)	5.04	2.16
6	100.0	0.07	< DL	82.4 (4.0)	0.79	< DL	61.7 (1.4)	2.68	< DL
7	100.0	0.06	< DL	98.7 (0.0)	0.06	< DL	100.4 (9.5)	0.08	< DL
8	100.0	0.10	< DL	88.8 (0.4)	2.35	0.47	79.5 (4.4)	5.37	2.21
9	100.0	0.09	< DL	92.4 (0.1)	0.65	0.45	85.9 (5.9)	2.10	2.24
10	NA	< DL	< DL	NA	< DL	< DL	NA	< DL	< DL
11	NA	< DL	< DL	NA	< DL	< DL	NA	< DL	< DL

**Standard deviations are indicated in parentheses when applicable.*

Capryol® 90 is a propylene glycol monocaprylate compound (molecular weight 202.29 g/mol) that can interact with SLD molecule (molecular weight 495.53) and release propylene glycol (molecular weight 76.1) to potentially form either an ester or amide of SLD and octanoic acid (molecular weights 621.5). LC-MS chromatography studies at 4.0 pH reported that impurity 1 a mass of 622.5 g/mol which can be a protonated form of the ester or amide between SLD and octanoic acid. Figure S1 describes the molecular weights and structures of: A) Silodosin (SLD), B) Capryol® 90 and the possibles chemical reactions to obtain the C) ester and D) amide forms between SLD and octanoic acid. Both ester and amide forms of SLD and octanoic acid were synthetized to study the chemical identity of impurity 1. Impurity 1 was identified as shown in Figure 2 in the work as the ester form of SLD and octanoic acid.

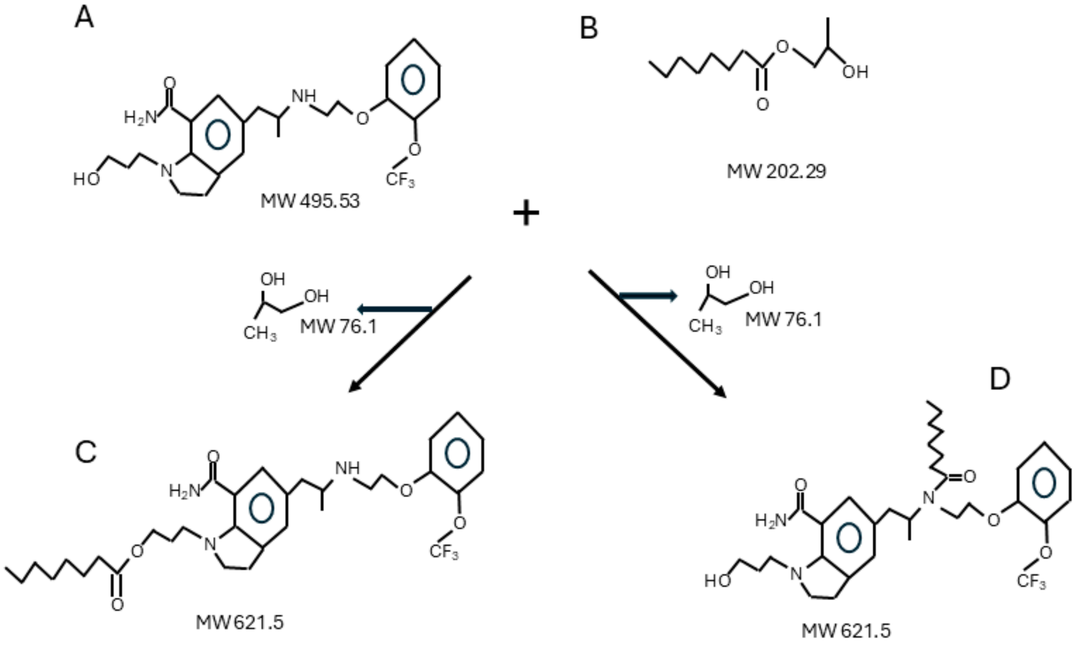

Figure S1. Chemical structures and molecular weights of SLD (A), propylene glycol monocaprylate (B), ester between SLD and octanoic acid (C), and the amide between SLD and the octanoic acid (D).

Long term ICH stability study

Table S2 shows the effects of packing material on the stability of SLD.

Table S2. Effect of packing material on stability of SLD (SD in parenthesis) depending on time exposition in months at the different storage conditions.

Temp/RH	Packing	0	1	3	6	9	12
40 ºC/75% RH	PVC / PVDC	100 (1.4)	101.1 (0.5)	97.4 (0.3)	92.7 (0.9)	-	-
	AquaBa®	100 (1.4)	99.5 (1.6)	99.5 (1.6)	96.9 (0.4)	-	-
30 ºC/75% RH	PVC / PVDC	100 (1.4)	100.1 (1)	99.9 (0.7)	99.2 (1.3)	-	-
	AquaBa®	100 (1.4)	97.8 (1.4)	99.6 (1.6)	99.2 (0.4)	99.6 (0.2)	98.2 (0.8)
30 ºC/65% RH	PVC / PVDC	100 (1.4)	100.7 (2)	99.9 (1)	99.5 (0.8)	-	-
	AquaBa®	100 (1.4)	98.1 (3.1)	100.6 (0.6)	99 (0.8)	99.4 (0.7)	99.1 (1)
25 ºC/60% RH	PVC / PVDC	100 (1.4)	99.2 (2.6)	100 (1.8)	100.1 (0.9)	-	-
	AquaBa®	100 (1.4)	97.2 (2.7)	101.2 (0.2)	99.3 (0.8)	99.9 (0.7)	99.4 (1.1)
